# Development of the Flu-PRO: a patient-reported outcome (PRO) instrument to evaluate symptoms of influenza

**DOI:** 10.1186/s12879-015-1330-0

**Published:** 2016-01-05

**Authors:** John H. Powers, M. Lourdes Guerrero, Nancy Kline Leidy, Mary P. Fairchok, Alice Rosenberg, Andrés Hernández, Sonja Stringer, Christina Schofield, Patricia Rodríguez-Zulueta, Katherine Kim, Patrick J. Danaher, Hilda Ortega-Gallegos, Elizabeth Dansie Bacci, Nathaniel Stepp, Arturo Galindo-Fraga, Kristina St. Clair, Michael Rajnik, Erin A. McDonough, Michelande Ridoré, John C. Arnold, Eugene V. Millar, Guillermo M. Ruiz-Palacios

**Affiliations:** 1 Clinical Monitoring and Research Program, Clinical Research Directorate, Leidos Biomedical Research, Inc., Frederick National Laboratory for Cancer Research, Frederick, MD, 21702 in support of the Collaborative Clinical Research Branch, Division of Clinical Research, National Institute of Allergy and Infectious Diseases, Bethesda, MD USA; 2 Instituto Nacional de Ciencias Médicas y Nutrición Salvador Zubirán, Mexico City, Mexico; 3 Evidera, Bethesda, MD USA; 4 Infectious Disease Clinical Research Program, Uniformed Services University, Bethesda, MD USA; 5 Henry M. Jackson Foundation for the Advancement of Military Medicine, Bethesda, MD USA; 6 Madigan Army Medical Center, Tacoma, WA USA; 7 Instituto Nacional de Enfermedades Infecciosas, Mexico City, Mexico; 8 Hospital General Dr. Manuel Gea González, Mexico City, Mexico; 9 San Antonio Health System, San Antonio, TX USA; 10 Camp Lejeune, Jacksonville, NC USA; 11 Naval Medical Center Portsmouth, Portsmouth, VA USA; 12 Walter Reed National Military Medical Center, Bethesda, MD USA; 13 Naval Health Research Center, San Diego, CA USA; 14 Naval Medical Center San Diego, San Diego, CA USA; 15 5601 Fishers Lane, Room 4D50, Rockville, MD 20852 USA

**Keywords:** Influenza, Symptoms, Patient-reported outcomes, Endpoints, Outcome assessments, Content validity

## Abstract

**Background:**

To develop content validity of a comprehensive patient-reported outcome (PRO) measure following current best scientific methodology to standardize assessment of influenza (flu) symptoms in clinical research.

**Methods:**

Stage I (Concept Elicitation): 1:1 telephone interviews with influenza-positive adults (≥18 years) in the US and Mexico within 7 days of diagnosis. Participants described symptom type, character, severity, and duration. Content analysis identified themes and developed the draft Flu-PRO instrument. Stage II (Cognitive Interviewing): The Flu-PRO was administered to a unique set of influenza-positive adults within 14 days of diagnosis; telephone interviews addressed completeness, respondent interpretation of items and ease of use.

**Results:**

Samples: Stage I: *N* = 46 adults (16 US, 30 Mexico); mean (SD) age: 38 (19), 39 (14) years; % female: 56 %, 73 %; race: 69 % White, 97 % Mestizo. Stage II: *N* = 34 adults (12 US, 22 Mexico); age: 37 (14), 39 (11) years; % female: 50 %, 50 %; race: 58 % White, 100 % Mestizo. Symptoms: Symptoms identified by >50 %: coughing, weak or tired, throat symptoms, congestion, headache, weakness, sweating, chills, general discomfort, runny nose, chest (trouble breathing), difficulty sleeping, and body aches or pains. No new content was uncovered during Stage II; participants easily understood the instrument.

**Conclusions:**

Results show the 37-item Flu-PRO is a content valid measure of influenza symptoms in adults with a confirmed diagnosis of influenza. Research is underway to evaluate the suitability of the instrument for children and adolescents. This work can form the basis for future quantitative tests of reliability, validity, and responsiveness to evaluate the measurement properties of Flu-PRO for use in clinical trials and epidemiology studies.

## Background

Influenza is a contagious, viral illness characterized by mild to severe symptoms that may lead to death [1]. In the United States (US), influenza and its complications result in approximately 111 million lost work days, $7 billion/year in sick days and lost productivity, 30 million clinic visits, 226,000 hospitalizations and 3,000 to 49,000 deaths annually [2–4]. Worldwide influenza and its complications result in an estimated 3–5 million cases of severe illness and approximately 250,000–500,000 deaths every year [5]. In patients who survive, much of the disability from influenza is due to bothersome symptoms that impair their ability to function.

Currently, there is no standardized, validated patient-reported outcome (PRO) measure for influenza and influenza-like symptoms developed according to accepted scientific standards [6–8]. A comprehensive, precise measure would facilitate understanding of the natural history and epidemiology of influenza as well as quantify its severity and recovery patterns of disease and its variability within and across populations. Combined with other baseline variables, quantification of symptoms would help define and standardize severity of illness measures for influenza. For prevention trials, a validated measure could be used to standardize and document the presence and severity of symptoms in patients who develop the disease. For treatment trials, a precise, disease-specific PRO symptom measure would be more sensitive to treatment effects, particularly time-to-event (symptom improvement) analyses. A standardized tool across studies would facilitate meta-analyses and comparisons of interventions.

The purpose of this study was to begin to develop a PRO instrument to standardize the assessment of influenza (Flu) symptoms, the Flu-PRO, by evaluating content validity using methods recommended by the US Food and Drug Administration (FDA) [6] and good research practices [7, 8].

## Methods

### Study design and setting

A two-stage qualitative instrument development methodology was followed, involving adults (≥18 years) with laboratory-confirmed influenza recruited through clinical sites in the US and Mexico. Stage I involved concept elicitation interviews with the target population to gather information regarding patient experience of influenza symptoms, including type, magnitude, expression, pattern of onset, and recovery. From this, a draft item pool and conceptual framework were developed for the new Flu-PRO instrument, including its content and structure (item phrasing, length, response options, recall, instructions). Stage II consisted of cognitive interviews to assess completeness, comprehension, and interpretability of the draft Flu-PRO instrument from the respondent’s perspective. Qualitative, semi-structured telephone interviews were utilized in both stages. Patients with laboratory-confirmed influenza were recruited from military treatment facilities or associated clinics in the US (six clinics in San Diego, CA; Portsmouth, VA; Tacoma, WA; San Antonio, TX; Jacksonville, NC; and Bethesda, MD), and the Mexico Emerging Infectious Diseases Clinical Research Network (LaRed) in Mexico, which consisted of three hospitals: Instituto Nacional de Ciencias Médicas y Nutrición Salvador Zubirán (Nutricion), Instituto Nacional de Enfermedades Respiratorias (INER), and Hospital General Dr. Manuel Gea. The protocol for both stages was approved by Institutional Review Boards at the Uniformed Services University and at both the US and Mexico sites.

### Stage I: concept elicitation interviews

The purpose of the concept elicitation interviews was to elicit participants’ descriptions of their influenza symptoms to inform the content and structure of the new measure. The following inclusion criteria were used to recruit potential participants from US sites: 1) aged 18 years or older; 2) eligible for care in Department of Defense facilities (DEERS eligible); 3) symptoms consistent with influenza-like illness (ILI): a) Fever with body temperature ≥100.4 °F [38 °C] AND respiratory symptoms OR b) Subjective fever AND respiratory Symptoms; 4) able to speak in English; and 5) able to provide written informed consent. Respiratory symptoms were defined as: cough, or sputum production, or shortness of breath, or chest pain, and/or sore throat.

Exclusion criteria were: 1) self-report of concurrent asthma or chronic obstructive pulmonary disease (COPD) exacerbation; 2) concurrent pneumonia symptoms with clinical evidence confirming bacterial pneumonia, i.e., pleural effusion or empyema, consolidated single lobar pneumonia documented by radiological testing with otherwise normal lung fields, or bacteremia with a pathogen known to cause pneumonia; 3) any other relevant condition that would interfere with participants’ ability to participate in a one-on-one telephone interview; and 4) complicated pregnancy.

For the Mexico participants, the following inclusion criteria were used to recruit participants: 1) aged 18 years or older; 2) enrolled in an existing influenza natural history study at the Mexico sites that included swab for influenza testing; 3) Spanish speaking; and 4) able to provide written informed consent. Exclusion criteria were: 1) any relevant condition that would interfere with their ability to participate in a one-to-one telephone interview; and 2) declined consent to having their interview audio-record interview.

All participants with ILI who met these criteria were enrolled and interviewed for this study; however, only those who were later shown to have a laboratory-confirmed influenza diagnosis were included in the analysis. This ensured the concepts elicited in developing the new questionnaire were from participants known to have confirmed influenza, rather than another ILI. Both inpatients and outpatients were eligible for inclusion. In both the US and Mexico, all participants’ influenza diagnostic procedure was performed as part of existing studies’ protocols and not specifically for the purpose of the Flu-PRO development study. These tests included rapid antigen testing (RAT) and real time reverse-transcription polymerase chain reaction (RT-qPCR) for influenza. Available results classified patients as influenza positive or negative, but did not further classify patients by type of influenza, as the medical literature poses the symptoms of influenza are similar across types of influenza and types of ILI.

Participants provided written informed consent at the recruitment site and were scheduled for a telephone interview with the study research team. A semi-structured interview guide was used to facilitate the interviews, with open-ended questions asking participants to describe their symptoms and characterize their nature, severity, onset, duration, variability, and indictors of recovery. Information on the importance or impact of symptoms was gathered to understand the relative importance of symptoms from a content validity perspective.

All interviews were conducted by trained researchers. US interviews were conducted in English by research staff from Evidera (Bethesda, MD) and interviews in Mexico were conducted in Spanish by staff from the LaRed network using the translated interview guide. At the end of the interview, participants were asked sociodemographic and clinical/health-related questions, with data used to characterize the sample. Interviews were transcribed verbatim, with the data cleaned and de-identified for analyses. Mexico interviews were translated by an external translation company (FACIT - Functional Assessment of Chronic Illness Therapy group; Chicago, IL) for analyses in English.

A coding dictionary was developed by examining initial transcript data to identify key themes and constructs participants expressed during the interviews. The software program ATLAS.ti (version 5.0) was used to facilitate the systematic analysis of data. All transcripts were then coded thematically and reviewed by a second analyst, with any discrepancies resolved through discussion with the primary or senior analyst. Symptoms that emerged from the interviews were tracked on a grid to monitor saturation, defined as the point at which additional sampling offered no new information [9]. US data from adult participants were collected and analyzed first and to the point of saturation. Data collected from Mexico were analyzed using the same coding dictionary, assessing symptoms reported and noting any differences with US results.

### Development of the Flu-PRO instrument

The multi-disciplinary Flu-PRO development team used results of the concept elicitation interviews to draft an English version of the Flu-PRO instrument, including items, instructions, recall period, and response options. This ensured the instrument was grounded in the words and phrases that patients with influenza commonly use to describe the symptoms they experienced. A translatability assessment was performed by an expert in PRO instrument translation and ePRO formatting to ensure translatability of words and phrases, and suitability of conversion to ePRO format.

An item definition dictionary and translation instructions were then developed and the draft Flu-PRO instrument was translated into Spanish using methods to ensure conceptual equivalence [10]. The steps involved in using this method were: 1) two forward translations by native speakers of Spanish; 2) one reconciled version of the two forwards by a third native speaker of Spanish; 3) one back translation of the reconciled version into English by an English speaker fluent in Spanish; 4) comparison of source text and back-translated text to identify possible discrepancies by FACIT staff; 5) review of the translations and FACIT comments by the bilingual language coordinator (LC) for Spanish; 6) finalization of the translations by the LC for Spanish; 7) quality review and resolution of any outstanding harmonization issues by FACIT; and 8) formatting by FACIT staff and proofreading of the final questionnaire by two professional translators who are native speakers of Spanish. Prior to its use in the cognitive interviews, investigators from Mexico reviewed the translated version to assure consistency with the words and phrases participants from Mexico used during the elicitation phase.

### Stage II: cognitive interviews

The purpose of the cognitive interviews was to assess subject comprehension and interpretability of the Flu-PRO, its ease of use, and completeness. The exclusion criteria and recruitment processes were the same as those used in the concept elicitation interviews outlined above. However, only participants with laboratory-confirmed influenza were enrolled within two weeks of diagnosis. A semi-structured interview guide was used to facilitate the cognitive interviews; questions assessed content coverage and participants’ understanding and interpretation of the Flu-PRO questionnaire (instructions, items, response options, recall period) with an open-ended question asking if there were any additional symptoms the person had experienced that were not covered in the questionnaire. As before, interviews were audio-recorded and transcribed verbatim. Sociodemographic and clinical/health-related questions were asked at the end of the interview with data used to characterize the sample. Mexico interviews were conducted in Spanish, transcribed, and translated into English for analyses. Item-by-item analyses were performed to assess clarity and consistency in interpretation across subjects, and to determine the need for adjustments or editorial changes before the instrument was finalized for quantitative evaluation.

## Results

### Stage I: concept elicitation interviews

#### Sociodemographic and clinical characteristics

The sociodemographic and clinical characteristics of the concept elicitation study sample are presented in Table 1. The average age of US participants was 38.4 years and 39.0 years in Mexico. Female participants composed 56.3 % and 73.3 % of the US and Mexico groups, respectively. In the US, 68.8 % of participants identified themselves as White. In Mexico, the most common racial identification was Mestizo (96.7 %). The time from first symptoms to interview was approximately 5.4 days in the US sample.

**Table 1:** Stage I and II: sample characteristics by country

Characteristics	Stage I (*N* = 46)		Stage II (*N* = 34)	
	US (*n* = 16)	Mexico (*n* = 30)	US (*n* = 12)	Mexico (*n* = 22)
Age (years)				
Mean (SD)	38.4 (18.8)	39.0 (13.5)	37.0 (14.1)	38.6 (11.4)
Range	19.0–84.0	22.0–67.0	22.0–65.0	23.0–55.0
Gender (N, %)				
Female	9 (56.3)	22 (73.3)	6 (50.0)	11 (50.0)
Ethnicity (N, %)				
Not Hispanic or Latino	15 (93.8)	0 (0.0)	11 (91.7)	0 (0.0)
Hispanic or Latino	1 (6.3)	30 (100.0)	1 (8.3)	22 (100.0)
Race (N, %)^a^				
American Indian or Alaska Native	3 (18.8)	0 (0.0)	0 (0.0)	0 (0.0)
Asian	2 (12.5)	0 (0.0)	1 (8.3)	0 (0.0)
Black or African American	2 (12.5)	0 (0.0)	4 (33.3)	0 (0.0)
White	11 (68.8)	1 (3.3)	7 (58.3)	0 (0.0)
Mestizo	1 (6.3)	29 (96.7)	0 (0.0)	22 (100.0)
Other	2 (12.5)	0 (0.0)	0 (0.0)	0 (0.0)
Educational status adults (N, %)				
Less than high school	0 (0.0)	4 (13.3)	0 (0.0)	0 (0.0)
High school graduate or equivalent	1 (6.3)	8 (26.7)	1 (8.3)	4 (18.2)
Some college or university, bachelor’s degree, or post graduate education	15 (93.8)	18 (60.0)	11 (91.7)	15 (68.2)
Other	0 (0.0)	0 (0.0)	0 (0.0)	3 (13.6)
Time interval to interview (mean days, SD)				
First notice symptoms to interview	5.4 (2.3)	<7	12.1 (4.3)	10.8 (9.9)
First notice symptoms to clinic visit	1.8 (1.2)	<7	2.7 (2.9)	4.0 (4.5)
Clinic visit to interview date	3.6 (2.0)	<7	9.4 (3.7)	6.8 (7.4)
Medications for influenza^a^(N, % Yes)				
None	1 (6.3)	0 (0.0)	2 (16.7)	0 (0.0)
Oseltamivir (Tamiflu)	8 (50.0)	15 (50.0)	6 (50.0)	9 (40.9)
OTC symptom relief	13 (81.2)	0 (0.0)	0 (0.0)	0 (0.0)
Other	0 (0.0)	0 (0.0)	9 (75.0)	19 (86.4)
Health conditions (N, %)				
None	9 (56.3)	18 (60.0)	4 (33.3)	13 (59.1)
Asthma or other lung condition	0 (0.0)	0 (0.0)	3 (25.0)	7 (31.8)
Depression	0 (0.0)	0 (0.0)	2 (16.7)	0 (0.0)
Diabetes	1 (6.3)	5 (16.7)	1 (8.3)	0 (0.0)
Heart attack/angina	0 (0.0)	0 (0.0)	1 (8.3)	0 (0.0)
Hypertension	1 (6.3)	0 (0.0)	4 (33.3)	2 (9.1)
Kidney disease	0 (0.0)	0 (0.0)	2 (16.7)	0 (0.0)
Lung disease	0 (0.0)	0 (0.0)	1 (8.3)	0 (0.0)
Osteoporosis	1 (6.3)	0 (0.0)	1 (8.3)	0 (0.0)
Raised cholesterol	1 (6.3)	0 (0.0)	2 (16.7)	0 (0.0)
Stomach ulcer	1 (6.3)	0 (0.0)	0 (0.0)	0 (0.0)
Tuberculosis	1 (6.3)	0 (0.0)	0 (0.0)	0 (0.0)
Other health conditions	6 (37.5)	0 (0.0)	4 (33.3)	3 (13.6)
Cardiovascular disorder	0 (0.0)	4 (13)	0 (0.0)	0 (0.0)

Abbreviations: *OTC* over the counter; ^a^Participants could choose more than one response

#### Themes (influenza symptoms)

Twenty symptom categories were identified from the US sample (Table 2). Symptom saturation was met, meaning no new themes or ideas were expressed by US participants during the last round of interviews. All themes were identified in both the US and Mexico participant interviews, except for two additional symptoms unique to the Mexico participants; “feeling warm/hot (not cold)” and “sun/light sensitivity”(Table 2). Although a number of participants mentioned signs (fever) or diagnoses (dehydration, ear infection), these specific concepts were not considered candidates for the Flu-PRO symptom measure since they did not directly reflect patients’ symptomatic experience, and symptoms of fever (warmth, chills, etc.) are already captured in other items. Symptom themes were evaluated relative to influenza symptoms reported in the literature and on two public health websites (Table 3). The elicited concepts were similar between inpatients and outpatients or between patients diagnosed by RT-qPCR or RAT.

**Table 2:** Concept elicitation: influenza symptoms by country

Symptom	Totals			
	US (*N* = 16)		Mexico (*N* = 30)	
	*N*	%	*N*	%
Cough	16	100 %	29	97 %
Throat symptoms	15	94 %	23	77 %
Headache	15	94 %	27	90 %
Weakness	14	88 %	26	87 %
Tired/lack of energy	13	82 %	21	70 %
Body pain	13	81 %	24	80 %
Sweating	13	81 %	18	60 %
General discomfort	12	75 %	24	80 %
Chills	11	69 %	23	77 %
Runny nose	11	69 %	18	60 %
Congestion	11	69 %	16	53 %
Gastrointestinal (nausea, vomit, diarrhea)	9	56 %	19	63 %
Chest (trouble breathing)	8	50 %	14	47 %
Sleep disruption	8	50 %	18	60 %
Loss of appetite	7	44 %	12	40 %
Sinus pressure	5	31 %	2	7 %
Hot and cold	4	25 %	13	43 %
Eye irritation	3	19 %	9	30 %
Dizzy/lightheaded	2	13 %	4	13 %
Sneezing	1	6 %	8	27 %
Feeling warm/hot	0	0 %	11	37 %
Sun/light sensitivity	0	0 %	6	20 %

**Table 3:** Influenza symptoms in the literature and professional websites

System symptom	Number of published papers (*n* = 32) or websites^a^ (*n* = 3) with this symptom (*N* = 35)
Upper respiratory	
Nasal congestion (e.g., obstruction, stuffiness)	14 (40.0 %)
Rhinorrhea (runny nose)	13 (37.1 %)
Sneezing	4 (11.4 %)
Sore throat	26 (74.3 %)
Hoarseness	3 (8.6 %)
Ear ache and/or pressure	4 (11.4 %)
Conjunctivitis	1 (2.9 %)
Sinus pain	1 (2.9 %)
Coryza	2 (5.7 %)
Nasal symptoms	2 (5.7 %)
Lower respiratory	
Cough	29 (82.9 %)
Dyspnea/difficulty breathing	4 (11.4 %)
Chest congestion	1 (2.9 %)
Chest discomfort	2 (5.7 %)
Systemic	
Headache	22 (62.9 %)
Muscle/joint aches and pains	25 (71.4 %)
Fatigue (weakness, tiredness, low energy)	16 (45.7 %)
Loss of or poor appetite	6 (17.1 %)
Vomiting	7 (20 %)
Nausea	3 (8.6 %)
Diarrhea	4 (11.4 %)
Digestive symptoms	2 (5.7 %)
Feverishness, chills, rigors, sweats	16 (45.7 %)
Dizziness	2 (5.7 %)
Malaise	3 (8.6 %)
Fatigue/weakness/tiredness/low energy	15 (42.9 %)
Signs	
Fever	32 (91.4 %)

^a^Websites: World Health Organization (WHO); National Institute for Allergy and Infectious Disease (NIAID); Centers for Disease Control (CDC)

#### Flu-PRO development

**Fig. 1:**
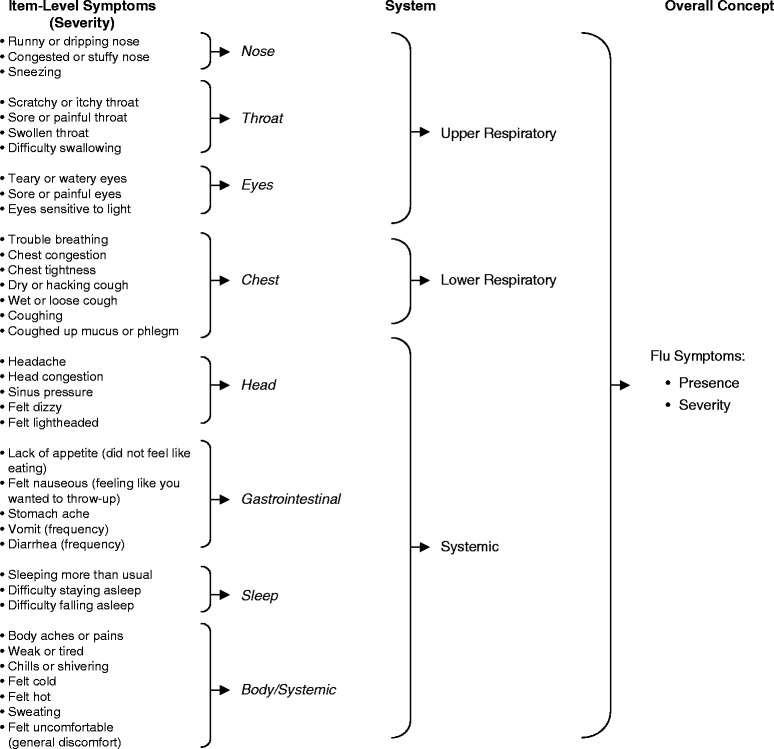
Flu-PRO draft conceptual framework

Symptoms were reviewed and discussed by the multi-disciplinary Flu-PRO team. Candidate items, response options, and structure (e.g., instructions and format) were developed, together with a conceptual framework for the instrument, using body systems as the organizational structure (see Fig. 1). For ease of use, a 5-point severity scale was selected, from “not at all” to “very much” for 32 items; 5-point frequency scales were used for vomiting and diarrhea (0 times to 4 or more times) and sneezing, coughing, and productive cough (never to always). Symptoms that were conceptually similar but worded differently by different participants were included in the item pool in order to subject the alternatives to qualitative and quantitative testing. For example, “scratchy or itchy throat”, “sore or painful throat”, “swollen throat”, and “difficulty swallowing” were all used to describe symptoms associated with a sore or inflamed throat. Rather than select one, all were included as candidate items for empirical testing. The instrument was formatted for ease of completion by paper-pen in a clinic or home setting, with minimal wording, check boxes, and gridlines. The format was also intended for ease of conversion to electronic format, including web-based or tablet, with no change in wording and minimal adjustments in appearance [11].

### Stage II: cognitive interviews

#### Sociodemographic and clinical characteristics

The sociodemographic and clinical characteristics of the study sample are presented in Table 1.

#### Understanding, saturation, and endorsement

Participants in both the US and Mexico samples had a good understanding of the instructions, recall period, response options, and the majority of the symptom items on the questionnaire. Participants considered the content and items to be relevant to their recent and/or previous influenza experiences. No new symptoms arose during the interviews, indicating symptom saturation had been reached during Stage 1.

As expected, participants identified symptom items they considered overlapping and/or highly related: “sore throat”, “swollen throat”, and “difficulty swallowing” (*n* = 5 US; *n* = 4 Mexico); ‘felt lightheaded” and “felt dizzy” (*n* = 5 US; *n* = 2 Mexico); and “head congestion”, “sinus pressure”, and “headache” (*n* = 4 US; *n* = 11 Mexico). Additionally, two participants from the US identified “sore and painful eyes” and “eyes sensitive to light” as overlapping. Participants from Mexico also identified the following overlapping/highly related symptoms: “sinus pressure”, “nasal congestion”, and “runny or drippy nose” (*n* = 10); “trouble breathing” and “nasal congestion” (*n* = 6); “chest tightness”, “pain with breathing or coughing”, and “muscular/general pain” (*n* = 5); and “body aches or pains” with “other” symptoms (*n* = 4). Because these comments were expected and raised by a relatively small number of people in this qualitative study, all of the items were retained for testing in a large, quantitative study, and flagged for additional consideration.

Stage III psychometric testing is currently underway and as part of this process, patients were timed in filling out the questionnaire. The total time to fill out the questions was approximately five minutes, showing low respondent burden and ease of use for clinical research studies.

**Fig. 2:**
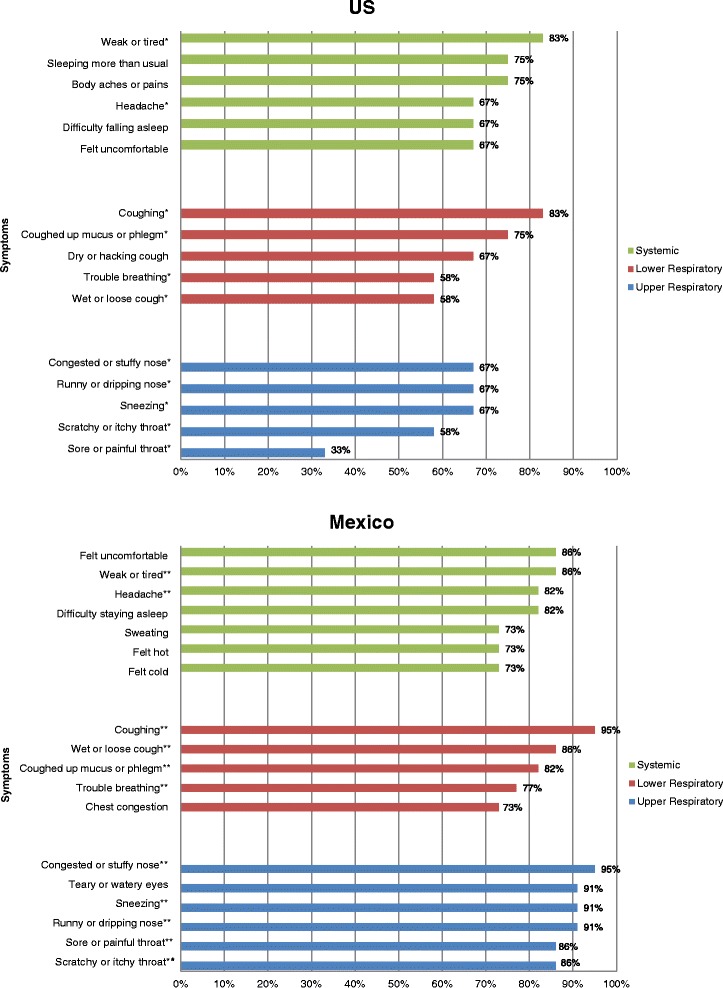
Cognitive interviews: Most frequently reported influenza symptoms, by system and country. *Symptoms also reported by participants with influenza in Mexico. **Symptoms also reported by participants with influenza in the US

**Fig. 3:**
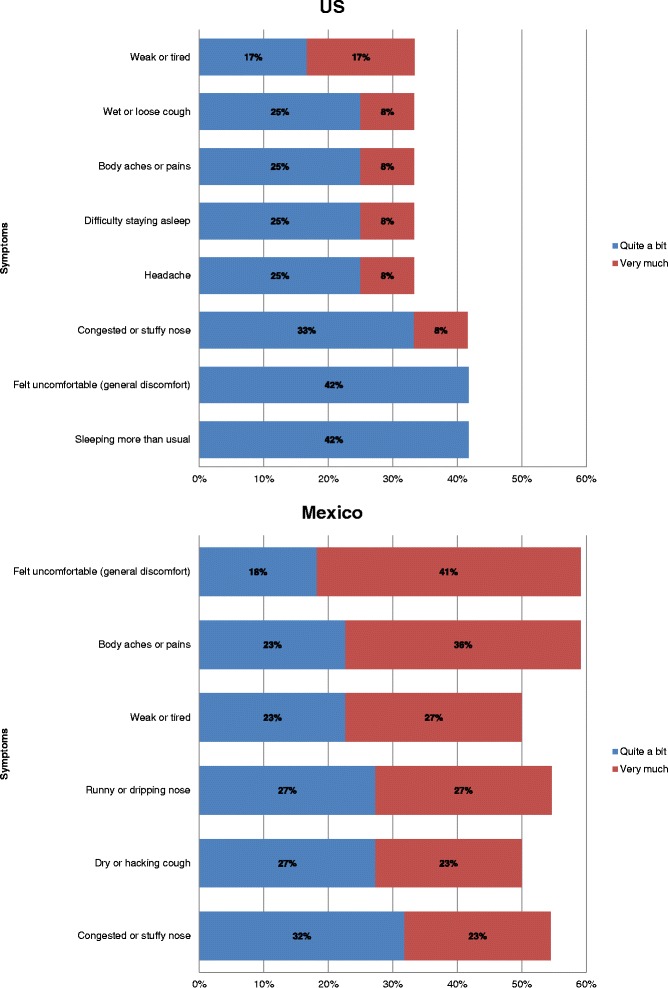
Cognitive interviews: FLU-PRO symptom Items rated as most severe, by country

Figure 2 shows the most frequently reported influenza symptoms by country, organized by body system. The symptoms participants rated as most severe, by country, are shown in Fig. 3.

## Discussion

The purpose of this study was to develop a new PRO influenza symptom measure (Flu-PRO) for adults using accepted methods to assure content validity. A comprehensive, precise, and standardized method for evaluating symptoms of influenza is important for natural history studies, tracking severity and burden of illness and clinical trials for prevention and treatment of influenza. Development of valid content in a standardized outcome assessment assures capturing a comprehensive assessment of patients’ symptom experiences with influenza, allowing both an accurate assessment of the burden of symptoms and comparison within and between studies.

Concept elicitation interviews amongst patients with confirmed influenza generated a range of symptoms and symptom descriptions that were consistent with symptoms described in the literature (Table 3). The results of this study showed a more comprehensive array of symptoms than those assessed in previous PRO instruments, including both systemic and gastrointestinal symptoms in addition to upper and lower respiratory symptoms. Results from Stage I were used in the development of candidate items, instructions, recall period, response options, and format for the Flu-PRO instrument. Care was taken to ensure that the items were phrased using words used by the interview participants. Cognitive interviews suggested excellent symptom coverage and completeness. Participants completed the Flu-PRO quickly and easily, and they understood the instrument content as intended. Clusters of symptoms considered similar, highly related, or redundant will be examined quantitatively during the item reduction phase of Stage III. The concepts elicited applied to both inpatients and outpatients with influenza, so the Flu-PRO could be useful in inpatients who can self-report. The elicited concepts did not differ by method of diagnosis (RAT or RT-qPCR), so the Flu-PRO should be generalizable regardless of the diagnostic test used.

Outcomes in clinical trials and epidemiologic studies of influenza have included clinician global assessments of signs and symptoms, symptom scales developed based on investigator opinion, and previously developed influenza symptom instruments. Two previous PRO symptom instruments specific to influenza have been described in the literature: the Influenza Symptom Severity Scale (ISS; [12]) and the symptom domain of the Influenza Intensity and Impact Questionnaire (FluiiQ™; [13]). The Influenza Wellbeing Scale (IIWS; [12]) has also been presented; however, this is a well-being scale rather than a symptom diary. The ISS is an 11-item measure that includes nasal congestion, sore throat, cough, aches and pains, fatigue, headache, feeling feverish, nausea, loss of appetite, disturbed sleep, and poor concentration, each rated on a 4-point severity scale ranging from 0 (absent) to 3 (severe) and summed to yield a total score. Content was based on information from a medical textbook of influenza rather than patients from the target population, and results of quantitative testing suggest noteworthy error in the total score (Cronbach’s alpha of 0.66, *N* = 52) [12].

The FluiiQ™ is a multi-dimensional instrument designed to improve on the ISS and IIWS, with domains measuring influenza symptoms (10 items), impact on daily activities (6 items), impact on emotions (4 items), and impact on others (5 items). Development of the FluiiQ™ included expert physician consultation (*n* = 7), focus group with college students with confirmed influenza (*n* = 6), and a concept mapping exercise with patients with a recent history of confirmed influenza in Australia (*n* = 16). The latter task asked participants to respond to a seeding statement asking how their recent flu episode had affected them and their lives. Cognitive interviews were performed with patients participating in focus groups. Content of the symptom domain of the FluiiQ™ includes headache, feeling feverish, body aches, fatigue, neck pain, interrupted sleep, loss of appetite, cough, sore throat, and nasal congestion. Quantitative testing (*N* = 311) showed two symptom factors, systemic (7 items) and respiratory (3 items), with internal consistency reliability (Cronbach’s alpha) levels of 0.85 and 0.48, respectively. These two scales and an overall symptom score (alpha = −0.82) were responsive to change over time and correlated with the impact domains of the FluiiQ™ [13]. Limitations of the symptom domain of the FluiiQ™ include the restricted number and type of symptoms assessed (e.g., absence of gastrointestinal symptoms, which have been noted as a part of the symptoms in newly emerging types of influenza), low reliability of the respiratory domain, and the embedded positioning within the larger FluiiQ™. We have also noted in this and previous work that the term “feverishness” relates to a multi-dimensional concept; therefore, the symptoms encompassed by the single word “feverishness” may not be clearly understood by patients. Our previous work showed that “fever” can relate to up to 16 different types of symptoms [14]. In the Flu-PRO, the various symptoms associated with fever are evaluated separately (warmth, chills, etc.) to increase understanding of respondents and allow for greater clarity on individual symptoms. Fever as a sign can be measured by body temperature measurements rather than PROs. Finally, the ISS and FluiiQ™ do not note the evaluation of inpatients with influenza, while inpatients were included in the development and evaluation of the Flu-PRO.

A focused, comprehensive PRO assessment of influenza symptoms for both inpatients and outpatients is needed in order to compare and contrast the type and severity of influenza symptoms across strains and sub-populations, and provide greater precision in clinical trials evaluating symptom severity and recovery as an efficacy endpoint. Flu-PRO is a symptom scale developed using current best scientific practices for development of PROs. The development process for the Flu-PRO assures that the symptoms captured are comprehensive and important to patients, and that the wording used is clearly understood by patients who would use the instrument. The concepts included in the Flu-PRO were endorsed by both inpatients and outpatients with laboratory-confirmed influenza by RAT and/or RT-qPCR.

While we enrolled patients who were ill enough to seek medical care, we did enroll patients with range of symptoms form mild to severe. Patients with less severe symptoms may have less intensity of symptoms but the concepts elicited are similar to those in more severe disease.

The next phase of the development process will be quantitative evaluation of the measure in English and Spanish-speaking adults in the US and Mexico, which is ongoing. Analyses will include item and factor analysis with item reduction and tests of score reliability, validity, and responsiveness. Research is also underway to examine the suitability of the Flu-PRO instrument for children and adolescents.

## Conclusion

This paper describes the qualitative methods used to optimize the content validity of the new Flu-PRO. Further research is required to test the quantitative properties of the items and scores generated by this new measure. When the latter is secured, the Flu-PRO will be available for use as a standardized method for evaluating symptoms of influenza in natural history studies and clinical trials.

## Abbreviations

COPD: Chronic obstructive pulmonary disease
DEERS: Defense Enrollment Eligibility Reporting System
FACIT: Functional Assessment of Chronic Illness Therapy
FDA: US Food and Drug Administration
Flu: Influenza
FluiiQ™: Influenza Intensity and Impact Questionnaire
IIWS: Influenza Wellbeing Scale
ILI: Influenza-like illness
INER: Instituto Nacional de Enfermedades Respiratorias
ISS: Influenza Symptom Severity Scale
LaRed: Emerging Infectious Diseases Clinical Research Network
LC: language coordinator
Nutricion: Instituto Nacional de Ciencias Médicas y Nutrición Salvador Zubirán
PRO: Patient-reported outcome
RAT: Rapid antigen testing
US: United States
